# Ubiquitin-protein ligase E3C promotes glioma progression by mediating the ubiquitination and degrading of Annexin A7

**DOI:** 10.1038/srep11066

**Published:** 2015-06-11

**Authors:** Si-Jian Pan, Shi-Kun Zhan, Wei-Zhong Ji, Yi-Xin Pan, Wei Liu, Dian-You Li, Peng Huang, Xiao-Xiao Zhang, Chun-Yan Cao, Jing Zhang, Liu-Guan Bian, Bomin Sun, Qing-Fang Sun

**Affiliations:** 1 Department of Neurosurgery, Rui-Jin Hospital, Shanghai Jiao-Tong University School of Medicine, Shanghai, 200025, P.R. China; 2 Department of Stereotactic and Functional Neurosurgery, Rui-Jin Hospital, Shanghai Jiao-Tong University School of Medicine, Shanghai, 200025, P.R. China; 3 Department of Neurology, Qinghai Provincial People’s Hospital, Xining, 810007, P.R. China

## Abstract

The ubiquitin-protein ligase E3C (UBE3C) belongs to the E3 ligase enzyme family and implicates in the ubiquitin-proteasome pathway, thus regulates physiological and cancer-related processes. Here, we investigated the expression and roles of UBE3C in glioma. We demonstrated that UBE3C was overexpressed in glioma tissues and cell lines. Inhibition of UBE3C expression in glioma cells significantly decreased cell migration and invasion *in vitro*. Mechanistically, we disclosed that UBE3C physically interacted with and ubiquitinated tumor suppressor gene annexin A7 (ANXA7), resulting in ubiquitination and degradation of ANXA7. Our results also revealed that increased UBE3C expression was accompanied by a reduction in ANXA7 protein expression in glioma tissues, but not ANXA7 mRNA. Importantly, the inhibition of ANXA7 expression in gliomas cells with UBE3C interference could rescue the cell invasion. Clinically, UBE3C overexpression significantly correlated with high-grade tumors (*p* < 0.05), poor overall survival, and early tumor recurrence. Thus, our data reveal that high UBE3C expression contributes to glioma progression by ubiquitination and degradation of ANXA7, and thus presents a novel and promising target for glioma therapy.

Gliomas, particularly glioblastoma multiforme (GBM), ranks as the deadliest human tumors, and are characteristically refractory to present treatments^1^,^2^. As such the overall median survival for patients with GBM is about 15 months according to population-based investigations^3^,^4^. To a certain extent, glioma progression contributed to accumulation of genetic mutations and aberrant gene expression, such as mutation of p53, phosphatase and tensin (PTEN) homologue, or retinoblastoma, and amplification of epidermal growth factor receptor or cyclin-dependent kinase-4^5^,^6^,^7^,^8^. However, the existing evidence is difficult to fully address the clinical pathological characteristics of glioma. Therefore, a deeper knowledge of the molecular pathogenesis of these tumors is of necessity to develop a new therapy for glioma.

Ubiquitin-dependent proteolysis is an important post-translational protein modification that regulates a set of critical cellular processes, including cell cycle progression, signal transduction, DNA repair, and apoptosis^9^,^10^. Ubiquitination mainly depends on the bioactivity and interaction of three enzymes: ubiquitin-activating enzyme (E1), ubiquitin-conjugating enzyme (E2), and ubiquitin ligase (E3). Among three enzymes, E3 ligase plays a crucial role in binding with specific substrate. E3 ligase contains two subtypes the homologous to E6-associated protein C terminus (HECT) domain and the RING finger domain E3 ubiquitin ligase^11^. Ubiquitin-protein ligase E3C (UBE3C, also known as KIAA10 or RAUL) is an established HECT domain E3 ubiquitin ligase that is able to catalyze a variety of unanchored polyubiquitin chains *in vitro*, and that targets the putative transcriptional factor TIP120B for proteolysis^12^,^13^. However, its physiological functions are largely unknown. Recently, Jiang *et al.*^14^ demonstrated that UBE3C may acted as a oncogene owing to its role in tumor development and progression, and thus it may serve as a potential therapeutic target in a subset of hepatocellular carcinoma patients^14^. In view of the roles of ubiquitin-proteasome pathway in cancer, it will make sense to study the roles and mechanisms of UBE3C in gliomas.

In the present study, we investigated the roles and mechanisms of UBE3C in glioma. We found that UBE3C was more highly expressed in glioma tissues compared to adjacent normal tissues, and elevated UBE3C expression resulted in the progression of glioma. UBE3C formed a complex with annexin A7 (ANXA7) and mediated ANXA7 protein degradation. Clinically, UBE3C overexpression correlated with poor survival of glioma patients. Thus, our results demonstrate that UBE3C may be a promising predictive biomarker for the poor prognosis of glioma, and may serve as an ideal therapeutic target for gliomas.

## Results

### UBE3C is overexpressed in glioma tissues

**Figure 1:**
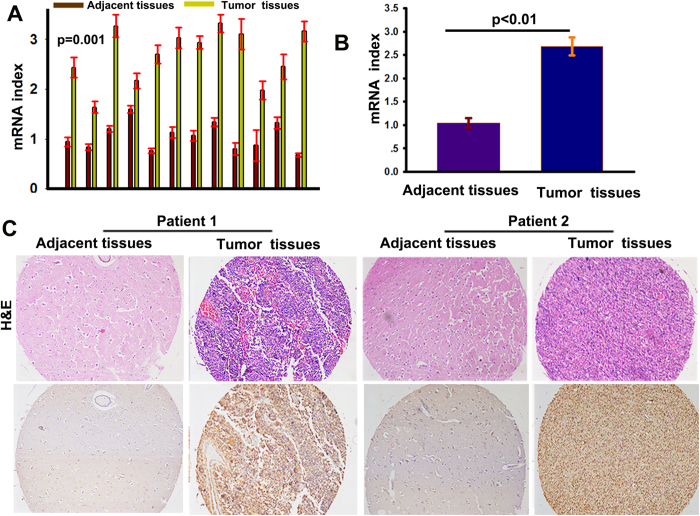
UBE3C overexpresses in gliomas tissues (A and B) The expression of UBE3C mRNA in gliomas samples and the adjacent nontumorous samples; (p < 0.01); **(C)** Hematoxylin-eosin staining and immunostaining of UBE3C in tumor and the adjacent nontumorous samples (bar = 50 μm).

UBE3C mRNA was highly expressed in glioma samples compared to the corresponding adjacent normal samples (Fig. 1A,B). There was a statistically significant difference in UBE3C expression between glioma and non-tumor samples (*p* = 0.01). After the identification of primary gliomas using hematoxylin and eosin staining, UBE3C protein expression was investigated by IHC in glioma tissues and adjacent non-tumor tissues. Immunoreactivity of UBE3C protein was observed in the cytoplasm of glioblastoma tumor cells (Fig. 1C), and very low levels of UBE3C expression were detected in non-tumor tissues compared to glioma tissues.

### Knockdown of UBE3C inhibits the migration and invasion of glioma cells

**Figure 2:**
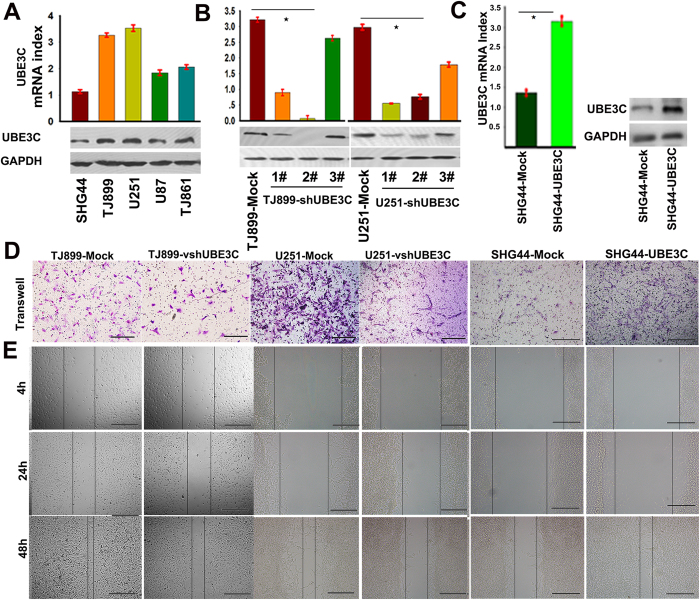
UBE3C interference decreased the migration and invasion of TJ899/U251 cells. RT-PCR and immunoblotting analyzed the expression of UBE3C in SHG44, U251, U87, TJ861 and TJ899 cells; **(B and C)** The expression of UBE3C were regulated in TJ899 and U251 cells by RNA interference; **(D)** Matrigel invasion assays showed that down-regulation of UBE3C was accompanied by a descend invasion of gliomas cells(bar = 100μm); **(E)** The scratch assay revealed that an conspicuous delay in the wound closure rate of TJ899/U251-vshRNA-UBE3C cells was found at 24 and 48h, compared with TJ899/U251-Mock cells(bar = 100 μm).

To investigate the role of UBE3C in glioblastoma cells, we first evaluated UBE3C expression in SHG44, U251, U87, TJ861, and TJ899 cells by *q*RT-PCR and immunoblotting. As shown in Fig. 2A, high levels of UBE3C were detected in U251 and TJ899 cells, whereas low levels were found in SHG44 cells. Then, U251 and TJ899 cells were transfected with pGMLV-GFP-vshRNA-UBE3C, and the efficiency of knockdown was evaluated by *q*RT-PCR and immunoblotting analysis (Fig. 2B). We used pGMLV-GFP-vshRNA-UBE3C-#2 in subsequent studies. At the same time, we also forced UBE3C expression in SHG44 cells (Fig. 2C). Matrigel invasion assays showed that decreased UBE3C expression reduced the invasiveness of TJ899 and U251 cells (Fig. 2D), while the up-reguletion of UBE3C in SHG44 cells raised the invasion ability of SHG44 cells. The wound-healing assay revealed an evident delay in the wound closure rate of TJ899-vshRNA UBE3C, U251-vshRNA UBE3C and SHG44-Mock cells at 24 and 48 h compared to TJ899-Mock, U251-Mock and SHG44-UBE3C cells (Fig. 2E and sFigs.1A, B and C).

### UBE3C forms a complex with ANXA7

**Figure 3:**
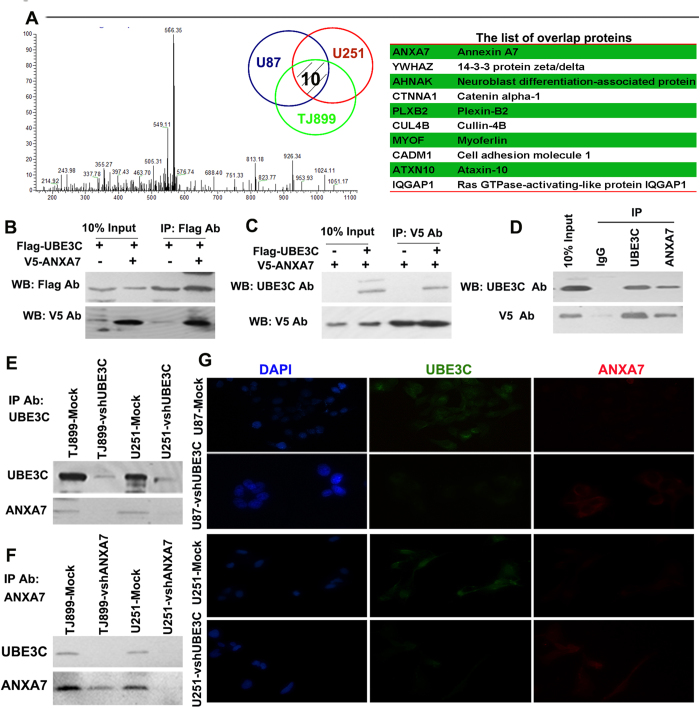
Proteomic Screen identifies UBE3C Interactes with ANXA7 to mediates ANXA7 Ubiquitin degradation Identification of the peptide from the UBE3C associated protein by Mass spectra, and there are ten proteins onerlap in U251, U87 and TJ899 cells, and the overlap protein was listed in the table; (B and C) Lysates of 293T cells transfected with plasmids encoding the Flag-UBE3C or V5-ANXA7 proteins were immunoprecipitated with the indicated antibodies and analyzed by immunoblotting with anti-Flag or V5 antibodies. **(D, E and F)** Co-IP assays demonstrated that UBE3C forms a complex with ANXA7 in gliomas cells; **(G)** Localization of UBE3C (green) and ANXA7 (red) in gliomas cells by immunofluorescence (Magnification, × 1,000);.

To determine the mechanism underlying UBE3C induction of glioma progression, we performed co-IP experiments followed by MS to determine the UBE3C-interacting proteins in U87, U251, and TJ899 cells (Fig. 3A). In total, 200, 251, and 241 proteins were identified as interacting with UBE3C in U87, U251, and TJ899 cells (supplementary Table 2), respectively. Of these, ten proteins overlapped (Fig. 3A). Recent literatures reported that ANXA7 was a suppressor gene in glioma^15^,^16^,^17^. Thus, we tried to further verify the relationship between UBE3C and ANXA7. We expressed Flag-tagged UBE3C and V5-tagged ANXA7 in 293T cells, followed by immunoprecipitation and immunoblotting analysis. In reciprocal co-IP assays using antibodies against Flag and V5, we determined that exogenous UBE3C forms a complex with ANXA7 in 293T cells (Fig. 3B,C). Furthermore, co-IPs using UBE3C antibodies pulled down ANXA7 (Fig. 3E, line 1 and 3), and in the reciprocal co-IP, UBE3C was pulled down using ANXA7 antibodies (Fig. 3F, line 1 and 3). Immunofluorescence also showed the co-localization of UBE3C and ANXA7 in glioblastoma cells (Fig. 3G). Together, these results strongly suggest that endogenous UBE3C form a physical complex with ANXA7.

### UBE3C promote the progression of gliomas by mediating ANXA7 degradation

**Figure 4:**
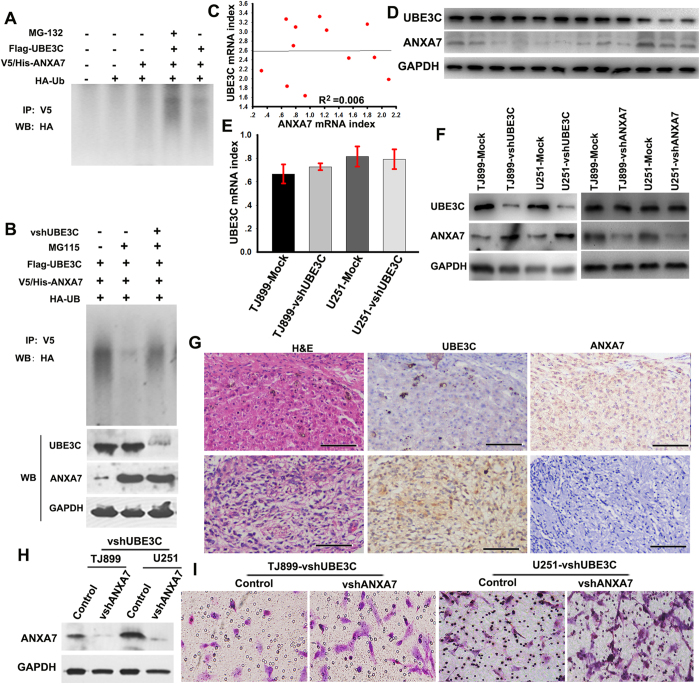
UBE3C promote gliomas progression by mediating ANXA7 degradation (A) The UBE3C on ANXA7 ubiquitylation in vivo. V5-ANXA7 and HA-Ub were co-transfected with Flag-UBE3C into 293T cells, and lysates of 293T cells were immunoprecipitated with the indicated antibodies and analyzed by immunoblotting using the anti-Flag, V5 and HA antibody, which detected poly-ubiquitylated ANXA7. **(B)** ANXA7 degradation is inhibited after UBE3C interference. **(C)** The correlation of UBE3C and ANXA7 mRNA in gliomas tissues; **(D)** The expression of UBE3C and ANXA7 proteins in gliomas tissues; **(E and F)** The forced loss of UBE3C up-regulated the ANXA7 protein, while not the ANXA7 mRNA; **(G)** Expression of UBE3C and ANXA7 protein was analyzed by IHC in gliomas tissues (bar = 200μm); **(H)** The expression of ANXA7 were regulated in TJ899 and U251-vshUBE3C cells by RNA interference; **(I)** Matrigel invasion assays showed that down-regulation of ANXA7 was accompanied by a descend invasion in TJ899 and U251-vshUBE3C cells (bar = 200μm).

We sought to determine whether UBE3C promotes AXNA7 proteasome-dependent proteolysis. Indeed, the ANXA7 protein could not be up-regulated in 293T cells with high level of UBE3C by transfecting the pGEX/V5-ANXA7, while its expression was enhanced by the proteasome inhibitor MG132 or UBE3C interference (Fig. 4A,B).

Next, we evaluated the correlation between UBE3C and ANXA7 mRNA and protein expression in 12 cases of glioma tissues by linear correlation analysis. Surprisingly, the results revealed that there was not significant correlation between UBE3C and ANXA7 mRNA (Fig. 4C, R2 = 0.006), whereas UBE3C protein expression had a strong negative correlation with ANXA7 (Fig. 4D). Among 12 cases analyzed, seven cases expressed high UBE3C expression and low ANXA7 (7/12, 58.33%), and three cases showed low UBE3C expression, but high ANXA7 expression (3/12, 25%). In UBE3C-modified glioma cells and the corresponding control cells, we found that downregulation of UBE3C led to increased ANXA7 protein expression, whereas it had no effect on ANXA7 mRNA expression, although knockdown of ANXA7 had little influence on UBE3C expression (Fig. 4E,F). IHC assay also showed that UBE3C protein expression positively correlated with low ANXA7 protein expression in glioma tissues, and vice versa (Fig. 4G). Importantly, the interference of ANXA7 in TJ899 and U251-vshUBE3C cells could restore the invasive ability of these cells (Fig. 4H,I), and the interference the UBE3C expression in SHG44 cells impaired SHG44 invasion (sFig. 3). These data suggest that UBE3C represent a bona fide endogenous ubiquitin E3 ligase that directly catalyzes the ubiquitination of ANXA7 and UBE3C promote gliomas progression by mediating ANXA7 degradation.

### UBE3C overexpression in gliomas is associated with poor patient prognosis

**Figure 5:**
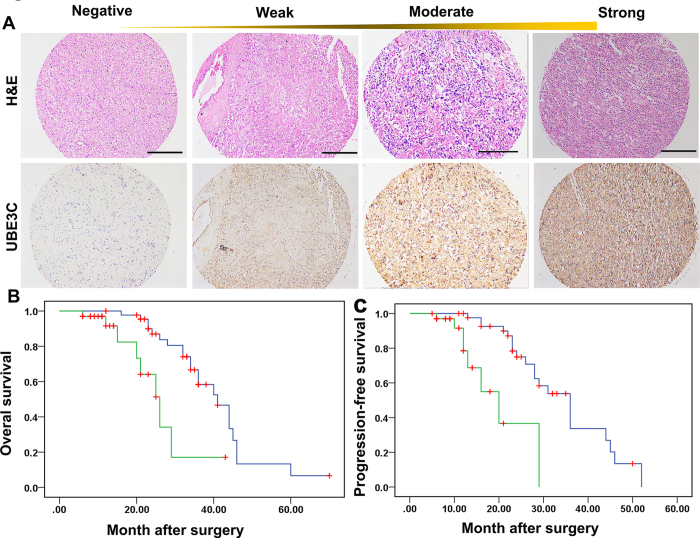
Overexpression of UBE3C was associated with the OS and PFS of gliomas Patients **(A)** Immunoreactivity of UBE3C protein was located in the cytoplasm of the gliomas cells (bar=50μm); **(B)** Prognostic significance assessed by Kaplan-Meier survival analysis, the OS and PFS of glioma patients compared by UBE3C. .

Positive UBE3C staining was detected in the cytoplasm of tumor cells (Fig. 5A). High levels of UBE3C were detected in 33 of the whole cohorts (33/80, 41.25%), and high UBE3C expression was more often detected in grade III and IV gliomas than in grade I and II gliomas. Furthermore, significant correlations were found between extent of surgery, chemoradiotherapy and UBE3C expression (Table 1). However, clinical characteristics, including age and sex were not significantly related to UBE3C expression.

All 80 patients had complete follow-up data. The median Overall survival (OS) was 23 months and average OS was 24.71 ± 13.24 months. The median Progression-free survival (PFS) was 16 months and average PFS was 18.28 ± 11.30 months. UBE3C expression also correlated with OS (*p* = 0.001, Fig. 5B) and PFS (*p* = 5.236 × 10^−5^; Fig. 5C). Univariate analysis showed that high tumor stage, positive glial fibrillary acidic protein (GFAP) expression, radiochemotherapy, total resection, and high UBE3C expression were predictors for OS and PFS. Age and sex had no prognostic significance for OS and PFS (Table 2). Individual clinicopathological features that showed significance by univariate analysis were adopted as covariates in a multivariate Cox proportional hazards model. Combined variables were further analyzed. Tumor grade and UBE3C expression were independent prognostic predictors of both OS (*p* = 0.014) and PFS (*p* = 0.004, respectively), while Tumor grade was an independent prognostic indicator of OS (*p* = 0.037, Table 3).

## Discussion

This study demonstrates that UBE3C expression is upregulated in glioma tissues compared to normal tissues, and its overexpression promotes the invasion and mobility of glioblastoma cells. Furthermore, we also elucidate that UBE3C forms a complex with ANXA7, and mediates its degradation. Clinically, we show that the elevated expression of UBE3C correlates with poor survival and early disease recurrence.

Now, several unspecific proteasome inhibitors as bortezomib and carfilzomib have shown clinical efficacy and been approved for clinical use. However, many scholars believe that inhibitors of more substrate-specific enzymes of degradation processes are needed^18^. Thus, our findings may be highly significant and relevant in the context of gliomas. On one hand, the results support the notion that UBE3C plays an important role in glioma progression. On the other hand, UBE3C mediating ANAX7 degradation may be used to develop the specific proteasome inhibitor for the treatment of gliomas.

The ubiquitin-proteasome system regulates crucial cellular functions, such as the cell cycle, DNA repair, cell signaling, and responses to hypoxia^10^,^19^. E3 enzymes play a key role in this process via the highly selective binding of protein substrates. The HECT domain E3 ligases possess intrinsic catalytic activity and directly catalyze the ubiquitination of substrate proteins, thereby performing pivotal roles in cellular homeostasis and biological signaling. Dysfunction of HECT E3 ligases frequently contributes to pathological disorders, including various tumors. An increasing number of tumor suppressor molecules have been identified as substrates of the HECT E3 ligases. As one of an important member of the HECT family of E3 ligases, E6AP could target tumor suppressor protein p53 for ubiquitin-mediated proteolysis to promote p53 degradation, contributing to the development of the majority of human cervical cancer^20^. Nedd4 was also reported to target a tumor suppressor PTEN that negatively regulated the phosphatidylinositol 3-kinase/AKT signaling pathway and acted as a substrate for proteasomal degradation, and thus associated with colorectal, bladder, and prostate carcinomas^21^. Additionally, genetic aberrations and altered expression of HECT E3 ligases were often seen in various human tumors^22^,^23^. In this study, we confirmed that UBE3C expression was markedly increased in glioma tissues compared to that in adjacent normal tissues. A more important finding from our study was that glioma patients with high UBE3C expression had poor prognosis. This finding may be also supported by the fact that glioma patients with aggressive clinicopathological features, such as high tumor grade, metastasis and poor differentiation had much higher UBE3C expression than those with low expression. This suggests that UBE3C may be a promising prognostic biomarker for gliomas.

Another important finding from present study was that UBE3C promoted glioma progression through formation of a complex with ANXA7 and ubiquitination and degradation of ANXA7. Co-IPs with MS and western blot analysis showed that UBE3C forms a complex with ANXA7. ANXA7 belongs to the group A annexin family, and is located on human chromosome 10q21 where multiple potential tumor suppressor genes exist^15^,^24^,^25^. ANXA7 protein is predominantly distributed in the membranes, and to a lesser extent in the nucleus, and contains a long N-terminal domain of ~200 amino acids rich in glycine, tyrosine, and proline residues^15^. ANXA7 is reportedly involved in exocytotic secretion and aggregation of chromaffin granules^25^. It causes liposome aggregation and forms classical voltage-gated Ca^2+^ channels in cellular and artificial membranes, which can be stabilized in long open states by GTP. Recently, ANXA7 was reported to be associated with tumors, including gliomas^15^. For example, the cancer genome atlas research network suggested that the ANXA7 might act as a tumor suppresser gene in glioblastoma, and another study found that haploinsufficiency of the tumor suppressor ANXA7 due to monosomy of chromosome 10 promotes glioblastoma cell tumorigenicity by augmenting EGFR signaling^16^,^26^. Recent study showed that the deregulation, loss of heterozygosity, and subcellular localization of ANXA7 were associated with the tumorigenesis, development, and metastasis of cancers through discretion of signaling pathways involving other tumor suppressors, DNA-repair, and apoptosis-related genes^23^. In reciprocal co-IP assays, we showed that UBE3C formed a complex with ANXA7 in 293T cells, and the significantly negative correlation of both proteins in glioma samples. These data indicate that UBE3C promotes glioma progression, which may result from the ubiquitination and degradation of ANXA7.

Our study clearly shows that high UBE3C expression contributes to glioma progression by ubiquitination and degradation of ANXA7, and thus presents a novel and promising target for future glioma therapies.

## Materials and methods

### Cell lines, lentiviral vectors, plasmids and other reagents

293T cell line, and five glioblastoma cell lines, SHG44, U87, U251,TJ861, and TJ889 (obtained from Chinese Academy of Sciences Shanghai Institutes for Biological Sciences Cell Resource Center, Shanghai, China) were cultured in Dulbecco’s Modified Eagle Medium (DMEM) supplemented with 10% fetal bovine serum at 37 °C in a humidified incubator under 5% CO_2_ conditions. Lentiviral-mediated pGMLV-GFP-shRNA-UBE3C/ANXA7 was manufactured by Shanghai Genomeditch Co. Ltd (Shanghai, China) to lessen UBE3C/ANXA7 expression in glioblastoma cells (abbreviated as U251- or TJ899-vshUBE3C/ANXA7 cells). The most effective shRNA targeting sequences were as follows: UBE3C, 5′-GCUUUUGUUCUCUCAGUACtt-3′; ANXA7, 5′-GTCAGAATTGAGTGGGAAtt-3′. Stable transfectant cells were verified by real-time polymerase chain reaction (*q*RT-PCR) and immunoblotting. The pcDNA3.1/Flag-UBE3C and pGEX/V5-ANXA7 plasmids were also constructed by Shanghai Genomeditech, Co. Ltd, and pcDNA3.1 and pGEX plasmids were used as a control. The transfections were performed as previously reported^27^,^28^.

The MG-132 (proteasome inhibitor) and MG-115 (reversible proteasome inhibitor) were bought from Beyotime Institute of Biotechnology (Shanghai, China).

### Patients, tissues collection, and tissue microarrays

Eighty samples were consecutively collected from the Department of Neurosurgery of Rui-Jin Hospital, Shanghai Jiao-Tong University (Shanghai, China) between January 2007 and September 2013. Patients were treated according to official therapeutic protocols, including surgical resection and subsequent chemoradiotherapy. A macroscopic total resection was performed in 56 of 80 patients (70%), and a partial resection was performed in 24 of 80 patients (30%). All patients underwent subsequent radiotherapy after surgical resection. Patients (64 of 80) received a median of four cycles (range, 1 ~ 9 cycles) of concurrent chemoradiotherapy with temozolomide. Surgical specimens were conventionally evaluated by two independent pathologists accordance to the WHO histological typing of central nervous system tumors. Following-up data were obtained by telephone interviews and outpatient department visits with the following-up rate being 100%. Freshly prepared glioma samples and non-tumor tissues (12 samples) were randomly chosen from 80 patients. The tissue microarrays were constructed as previously described^29^ and the simple construction method was included in supplementary methods^30^,^31^.

### Ethic statement

Written informed consent was obtained from each subject or legal guardian and signed by subjects and legal guardians prior to participation in the study. The research was approved by the Medical Ethical Committee of Shanghai Jiao-Tong University School of Medicine, and all experimental methods were carried out in accordance with approved guidelines of Shanghai Jiao-Tong University School of Medicine.

### RNA isolation and qPCR

Total RNA was extracted from gliomas cells and tissues using TRIZOL (Invitrogen, Carlsbad, CA). Total RNA (1 μg) was used to synthesize first-strand cDNA. The 2 μL cDNA was then used as a template for PCR amplification using the ABI PRISM 7900 Sequence Detection System (Applied Biosystems, Foster City, CA, USA) with power SYBR Green PCR Master Mix (TaKaRa, Dalian, China) GAPDH was used as an internal control, and the primers are included in Supplementary Table 1^−ΔCt^ [ΔCt = Ct (UBE3C/ANXA7) − Ct (GAPDH)] was calculated and used as an indication of relative expression levels. The experiments were repeated in triplicate.

### Immunoblotting, immunohistochemistry (IHC) and immunofluorescence

Immunoblotting, IHC and immunofluorescence were performed as previously reported. For immunoblotting, ployclonal mouse anti-human UBE3C (1:1,000; Abnova, Walnut, CA USA) was used to detect the expression of UBE3C, rabbit polyclonal to annexin VII-C-terminal was used to detect ANXA7 expression (1:2,500; Abcam, Cambridge, MA, USA), and anti-GAPDH (1:5,000; Chemicon, Temecula, CA, USA) was used as an internal control. For fluorescence, the UBE3C (1:300) and ANXA7 (1:200) antibodies were used. Nuclei were counterstained with 4,6-diamidino-2-phenylindole (DAPI, Vector Laboratories, Burlingame, CA, USA). The IHC staining intensity was scored as previously reported^14^.

### The scratch and invasion assays

The scratch and invasion assays were performed as previously reported^32^. At 4, 24, and 48 h after scratching, the healing margins were photographed, and then compared between the experimental and control groups. Invasion assay was performed using 24-well plates. Glioblastoma cells were cultured in the upper chamber in the insert with a concentration of 1 × 10^5^ cells per well. After 24 h, the inserts were removed from 24-well plates. After washing, the upper matrigel layer was erased, and the membrane were fixed in 4% paraformaldehyde and stained with Giemsa. Three separate experiments were performed.

### Immunoprecipitation

Immunoprecipitation was performed as previously described^31^. Briefly, 5 × 10^8^ glioblastoma cells were lysed by incubating at 4 °C for 2 h with 1 ml lysis buffer (10 mM Tris, pH 7.5, 150 mM NaCl, 1 mM CaCl_2_, 1 mM MgCl_2_, 1% Brij 97). Lysates were centrifuged at 16,000 × *g* for 15 min, and the supernatant was pre-cleared with a combination of protein A/G-agarose beads (Amersham Biosciences) pre-coated with BSA and goat serum (Sigma) for 2 h at 4 °C. Then, the lysate was incubated with a specific antibody coupled to protein A/G-agarose beads for 2 h at 4 °C. Immune complexes recruited by the beads were then washed four times with lysis buffer, eluted using 1% Triton X-100, acetone-precipitated, and resolved on 10% SDS-PAGE gels under non-reducing conditions. Finally, proteins were detected by immunoblot analysis using specific antibodies or liquid chromatography tandem mass spectrometry (LC-MS/MS).

### LC-MS/MS and database search

The immunoprecipitated material, gathered from the U87, U251, and TJ899 cells, was analyzed by LC-MS/MS and the ComPass program according to previous reports^32^,^33^.

All MS/MS data were analyzed by SEQUEST (v.28, Bioworks 3.3 software package, Thermo Electron) against the Human International Protein Index (IPI) database (IPI human v3.45 FASTA with 71983 entries).

### Ubiquitination Assay

The ubiquitination assays were performed according to previous reports^31^,^32^. 293T cells were grown to 60% density in 6-well plates. The cells were transfected with V5-ANXA7, HA-ubiquitin, Flag-UBE3C. After 48 hours posttransfection, cells were treated with 20 Μm of the proteasomal inhibitor MG132. Then anti-HA precipitates from the cell lysates were analyzed by immunoblotting using anti-V5 and anti-Flag antibodies.

### Statistical analysis

All statistical analyses were performed with SPSS 19.0 software (SPSS, Chicago, IL, USA). Values are described as mean ± standard deviation. Student’s *t*-test was used for comparisons between groups. OS was defined as the interval between gliomas resection and death or the date of last follow-up. PFS was calculated from resection to first detection of recurrence or progression by magnetic resonance imaging. For univariate analysis, progression-free survival and OS were evaluated by the Kaplan-Meier method, and differences were assessed through the log-rank test. Multivariate analysis was assessed via Cox’s proportional hazards regression model. The *p*-values less than 0.05 were considered statistically significant.

## Additional Information

**How to cite this article**: Pan, S.-J. *et al.* Ubiquitin-protein ligase E3C promotes glioma progression by mediating the ubiquitination and degrading of Annexin A7. *Sci. Rep.* **5**, 11066; doi: 10.1038/srep11066 (2015).

## Supplementary Material

Supplementary Information

## Figures and Tables

**Table 1:** The relationship of UBE3C expression with 80 Patients’ clinical characteristics.

**Characteristic**	**Low UBE3C expression^†^ (N = 47)**	**High UBE3C expression^†^ (N = 33)**	***p* value**
Age			
<50 y	28	13	
≥50 y	19	20	0.292
Sex			
Female	22	17	
Male	25	16	0.427
Tumor grade			
I-II	32	4	
III-IV	15	29	0.011^*^
Total resection			
Party	26	9	
Total	21	24	**4.57E-5**
chemoradiotherapy			
−	19	5	
+	28	28	**0.015**
GFAP			
+	21	7	
−	26	26	**0.007**

**Abbreviations and Note:** †, The staining intensity was graded as follows: Negative, 0-25% staining; Weakly, 26-50% staining; moderate, 51-75% staining; and strong, 76-100% staining = 4. UBE3C^high^, ≥51% of tumor section, and UBE3C^low^, <51%; ^*^Fisher’s Exact Test.

**Table 2:** Univariate analysis of factors associated with gliomas survival and recurrence.

	**OS**		**PFS**	
**Variables**	**Hazard ratio (95% CI)**	***p* value**	**Hazard ratio (95% CI)**	***p* value**
Sex (Male *vs.* Female)	0.782 (0.371–1.641)	0.516	0.827(0.386–1.775)	0.626
Age (years) (≤51 *vs.* >51)	0.903 (0.424–1.922)	0.791	0.947 (0.450–1.990)	0.885
Tumor grade (I/II *vs*. III/IV)	4.727 (1.821–12.274)	**0.002**	**6.796 (2.308**–**20.012)**	**5.054E-4**
GFAP (+ *vs*. −)	3.302 (1.330–8.202)	**0.011**	2.009 (0.842–4.792)	**0.118**
Total resection (+ *vs*. −)	3.083 (01.390–6.838)	**0.007**	**3.324(1.471**–**7.531)**	**0.004**
Radiochemotherapy (+ *vs.* −)	2.078 (0.960–4.501)	0.064	1.585 (0.724–5.938)	**0.242**
UBE3C^low^ *vs.*UBE3C^high^	4.032 (1.633–9.953)	**0.005**	**5.999 (2.295**–**15.681)**	**0.001**

**Abbreviations and Note:** OS, Overall survival; PFS, Progression-free survival; 95% CI, 95% confidence interval; HR, Hazard ratio; Cox proportional hazards regression model.

**Table 3:** Multivariate analysis of factors associated with OS and PFS.

	**Hazard ratio (95% CI)**	***p* value**
**OS**^**†**^		
Tumor grade (I-II *vs*. III-IV)	2.907 (1.066–7.929)	**0.037**
Total resection (+ *vs*. −)	2.278 (0.872–5.956)	0.093
GFAP(+ *vs.* −)	2.083 (0.855–5.076)	0.106
UBE3C^low^ *vs.*UBE3C^high^	2.982 (0.942–9.435)	0.063
**PFS**^**†**^		
(I-II *vs*. III-IV)	5.113 (1.737–15.053)	**0.001**
Total resection (+ *vs*. −)	2.018 (0.748–3.324\)	0.078
UBE3C^low^ *vs.*UBE3C^high^	3.64 (0.989–13.407)	**0.004**

**Abbreviations and Note**: 95% CI, 95% confidence interval; Multivariate analysis, Cox proportional hazards regression model. †, Variables were adopted for their prognostic significance by univariate analysis with forward stepwise selection (Forward, likelihood ratio). Variables were adopted for their prognostic significance by univariate analysis (*p*<0.05).
