# Correction: Ubiquitin-protein ligase E3C promotes glioma progression by mediating the ubiquitination and degrading of Annexin A7

**DOI:** 10.1038/s41598-026-70397-6

**Published:** 2026-09-15

**Authors:** Si-Jian Pan, Shi-Kun Zhan, Wei-Zhong Ji, Yi-Xin Pan, Wei Liu, Dian-You Li, Peng Huang, Xiao-Xiao Zhang, Chun-Yan Cao, Jing Zhang, Liu-Guan Bian, Bomin Sun, Qing-Fang Sun

**Affiliations:** 1 Department of Neurosurgery, Rui-Jin Hospital, Shanghai Jiao-Tong University School of Medicine, Shanghai, 200025 People’s Republic of China; 2 Department of Stereotactic and Functional Neurosurgery, Rui-Jin Hospital, Shanghai Jiao-Tong University School of Medicine, Shanghai, 200025 People’s Republic of China; 3 Department of Neurology, Qinghai Provincial People’s Hospital, Xining, 810007 People’s Republic of China

Correction to: *Scientific Reports* https://doi.org/10.1038/srep11066, published online 11 June 2015

This Article contains errors in Fig. 2.

During the Figure assembly process, representative images of Invasion in U251-Mock were placed in Fig. 2D and representative images of Scratch in TJ899-vshUBE3C (4h) and SHG44-Mock (48h) were placed in Fig. 2E.

The correct Fig. 2 and accompanying legend appear below.

**Fig. 2:**
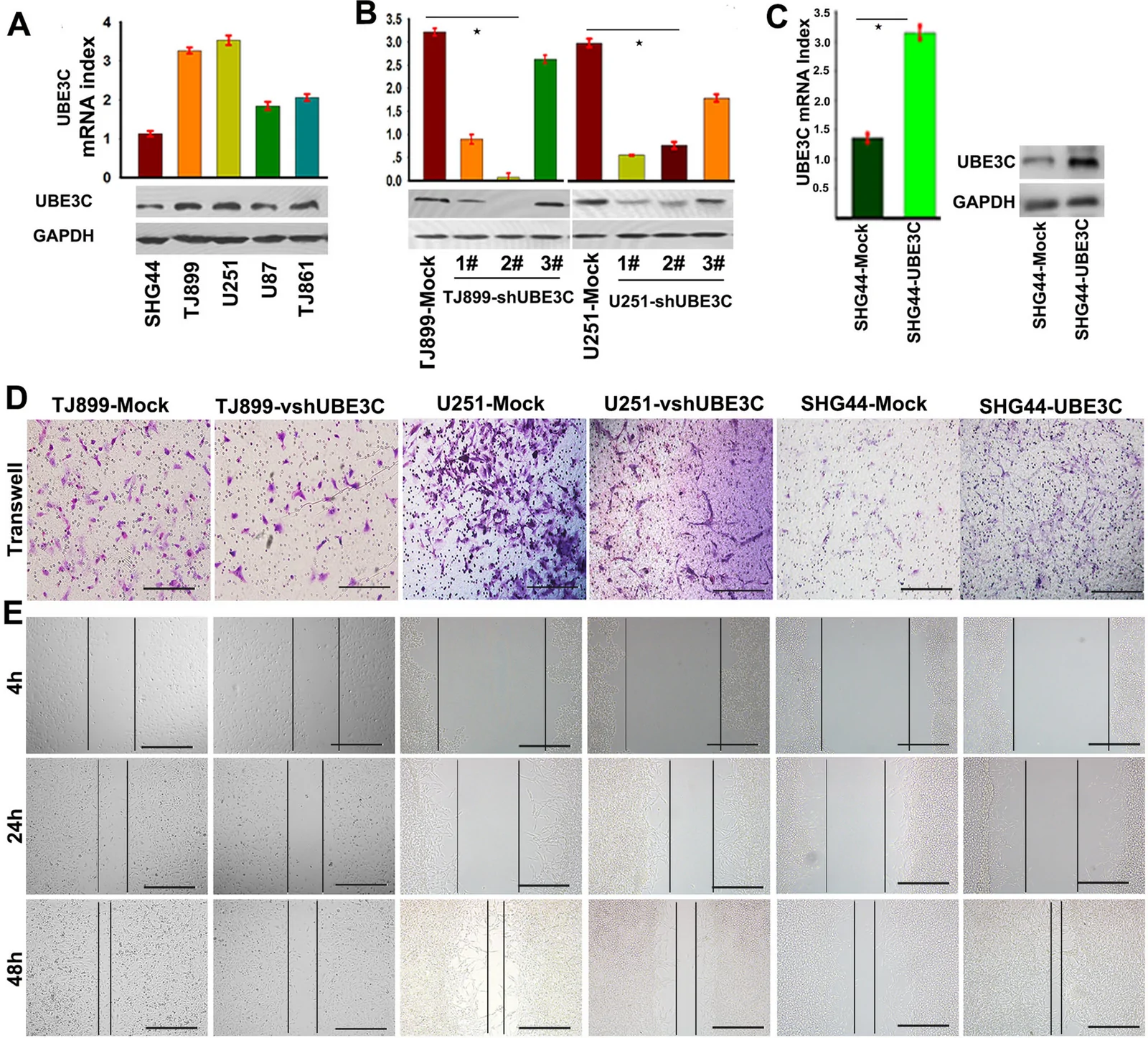
UBE3C interference decreased the migration and invasion of TJ899/U251 cells. RT-PCR and immunoblotting analyzed the expression of UBE3C in SHG44, U251, U87, TJ861 and TJ899 cells; (**B** and **C**) The expression of UBE3C were regulated in TJ899 and U251 cells by RNA interference; (**D**) Matrigel invasion assays showed that down-regulation of UBE3C was accompanied by a descend invasion of gliomas cells(bar = 100μm); (**E**) The scratch assay revealed that an conspicuous delay in the wound closure rate of TJ899/U251-vshRNA-UBE3C cells was found at 24 and 48h, compared with TJ899/U251-Mock cells(bar = 100 μm).

